# Karyotypic variation in the Andean rodent *Phyllotis xanthopygus* (Waterhouse, 1837) (Rodentia, Cricetidae, Sigmodontinae)

**DOI:** 10.3897/CompCytogen.v8i4.8115

**Published:** 2014-12-19

**Authors:** Carolina Alicia Labaroni, Matías Maximiliano Malleret, Agustina Novillo, Agustina Ojeda, Daniela Rodriguez, Pablo Cuello, Ricardo Ojeda, Dardo Martí, Cecilia Lanzone

**Affiliations:** 1 Laboratorio de Genética Evolutiva, IBS (CONICET-UNaM), Félix de Azara 1552, CP3300 Posadas, Misiones, Argentina; 2 Grupo de Investigaciones de la Biodiversidad (GiB), IADIZA, CONICET, CCT-Mendoza, CC 507, CP5500 Mendoza, Argentina, Instituto Argentino de Zonas Áridas

**Keywords:** Chromosomes, constitutive heterochromatin, fluorochromes, genetic variability, mammals

## Abstract

*Phyllotis xanthopygus* (Waterhouse, 1837) is an Andean rodent endemic to South America. Despite its wide geographical distribution in Argentina, few individuals have been studied on the cytogenetic level and only through conventional staining. In this work, chromosome characterization of Argentine samples of this species was performed using solid staining, C-banding and base-specific fluorochromes. Twenty two specimens were analyzed, collected in the provinces of Jujuy, Catamarca, and the north and south of Mendoza. All studied specimens showed 2n=38, having mostly the bi-armed autosomes, metacentric or submetacentric. Fundamental Number varied between 70 and 72. These changes were due to the presence of chromosome heteromorphisms in individuals from southern Mendoza and Jujuy. C-banding revealed pericentromeric blocks of constitutive heterochromatin in most chromosomes. Acrocentric chromosomes involved in heteromorphisms showed high variation in the amount of heterochromatin within and among populations. Additionally, banding with fluorochromes (DAPI and chromomycin A_3_) revealed homologous localization of AT and GC rich regions among chromosomes of the different populations analyzed. Comparisons among heteromorphic pairs suggested, however, that the variation might be the result of complex chromosome rearrangements, involving possibly amplifications and/or deletions of heterochromatic segments. These results are in accordance with molecular studies that indicate genetic variability within and among the populations of this taxon.

## Introduction

The sigmodontine rodents constitute one of the most diverse and broadly distributed Neotropical mammalian groups. Within the subfamily Sigmodontinae, the genus *Phyllotis* Waterhouse, 1837 (leaf-eared mice, or pericores) includes about 13 species and its geographic range extends from Ecuador to southern Argentina (Musser and Carleton 2005). *Phyllotis xanthopygus* has a broad distribution in Peru, Bolivia, Chile, and Argentina. Characterized as a montane species, it occupies a variety of habitats among which are grassland and desert regions (Kramer et al. 1999). It is distributed over an extensive elevation gradient ranging from high elevations in the central Andes (5000 m.a.s.l) to sea level. This distribution pattern provides an excellent natural experiment for exploring the effects of mountain topography on phylogeography and speciation (Albright 2004).

The taxonomic history of *Phyllotis xanthopygus* has been intertwined with that of *Phyllotis darwini* (Waterhouse, 1837), principally in the area of Central Chile where populations of *Phyllotis xanthopygus* were assigned to *Phyllotis darwini* at the species level (Spotorno and Walker 1983, Walker et al. 1984, Kramer et al. 1999). However, the specific recognition of *Phyllotis xanthopygus* is supported by studies based on morphometric, chromosomal and molecular differences (Spotorno and Walker 1983, Walker et al. 1984, Steppan et al. 2007). Six subspecies of *Phyllotis xanthopygus* have historically been recognized: *Phyllotis xanthopygus chilensis* Mann, 1945, *Phyllotis xanthopygus posticalis* Thomas, 1912, *Phyllotis xanthopygus ricardulus* Thomas, 1919, *Phyllotis xanthopygus rupestris* (Gervais, 1841), *Phyllotis xanthopygus vaccarum* Thomas, 1912 and *Phyllotis xanthopygus xanthopygus* (Waterhouse, 1837), which have typically been described by morphological traits (Steppan 1993). The species *Phyllotis limatus* Thomas, 1912 and *Phyllotis bonariensis* Crespo, 1964 are embedded in a *Phyllotis xanthopygus* complex within the *Phyllotis darwini* species group (Steppan et al. 2007). Data from phylogenetic analysis of both mitochondrial and nuclear DNA support that the *Phyllotis xanthopygus* complex is characterized by deep divergences and high genetic diversity (Kim et al. 1998, Steppan et al. 2007).

The genus *Phyllotis* has a high degree of karyotypic diversification. The diploid number shows variations from maximal 2n=68 in *Phyllotis osilae* Allen, 1901 to minimal 2n=38 shared by several species of the genus. The chromosome complement of *Phyllotis xanthopygus* is 2n=38 with all chromosomes biarmed (Pearson and Patton 1976, Spotorno et al. 2001). However, some departures from such a “common” karyotype have been reported. Pearson and Patton (1976) using routine solid staining described a karyotype with a single acrocentric element in two specimens of *Phyllotis xanthopygus* from the Central Andes. This was interpreted as a possible pericentric inversion. These changes represent one of the most frequent chromosome rearrangements and, consequently, a very common source of karyotypic variation in rodents (Patton and Sherwood 1983).

Constitutive heterochromatin (CH) is a feature that is often variable among *Phyllotis* and other rodents (Walker et al. 1991). Studies of related *Phyllotis* species have shown substantial differences in amount of CH. Comparative C-banding studies performed in three subspecies of *Phyllotis xanthopygus* from Chile showed intra and interspecific CH variation. An important difference was found between *Phyllotis xanthopygus xanthopygus*, which exhibits most autosomes with very tiny pericentromeric C-bands, and *Phyllotis xanthopygus rupestris* and *Phyllotis xanthopygus vaccarum*, with large pericentromeric C-bands on all their autosomes. On the other hand, the G-banding patterns of these subspecies were similar, with the exception of the sex chromosomes from *Phyllotis xanthopygus xanthopygus* (Walker et al. 1984, 1991). Additionally, patterns of fluorescent bands, which identify sequences rich in AT and GC base pairs, are very informative and have been useful for determining chromosome homologies comparable to G- and R-banding respectively. Moreover, they provide information about the distribution of these sequences within the genome (Veyrunes et al. 2007).

Studies of banding patterns are important to establish karyotype homology and specify the chromosome rearrangements accompanying processes of taxonomic diversity and karyotype evolution in a taxon. Differential chromosome banding in *Phyllotis* species has been published only for Chilean specimens (Walker et al. 1991). Previous cytogenetic studies on Argentine populations of *Phyllotis xanthopygus* are very scarce and used routine techniques only (Pearson and Patton 1976, Albright 2004). We are presenting here a wider intraspecies spectrum of data on the karyotype of this species which will allow us to assess the distribution of chromosomal variation in *Phyllotis xanthopygus* along provinces of Argentina.

## Material and methods

**Figure 1.**
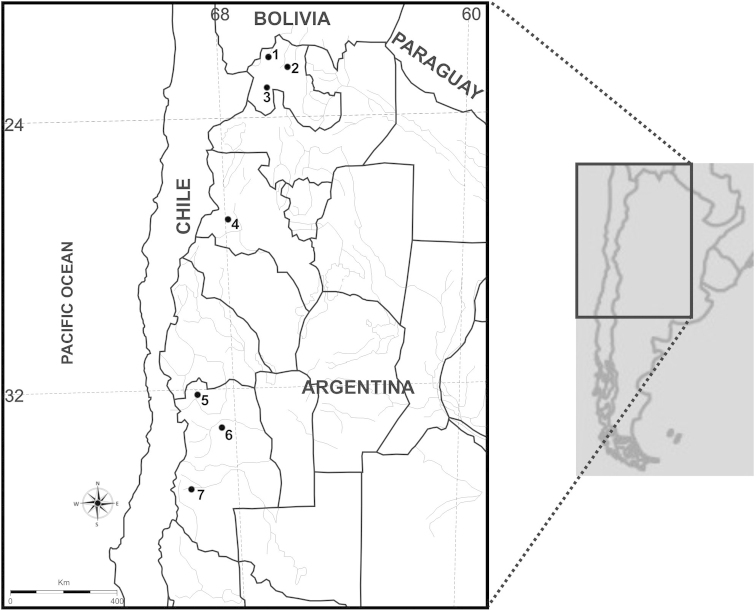
Map showing collection sites of *Phyllotis xanthopygus* in 3 provinces of Argentina: Jujuy, **1**) Loma Blanca, **2**) Abra Pampa, **3**) Susques; Catamarca, **4**) Cortaderas and Pastos Largos; Mendoza, **5**) Uspallata, **6**) Las Heras and Quebrada del Toro, **7**) Malargue. Localities **5** and **6** correspond to the north and **7** to the south of Mendoza province, respectively.

For chromosome study, 22 individuals of *Phyllotis xanthopygus* were collected across the Puna and Monte desert biomes. Fig. 1 shows north-south distribution of 7 of 9 listed below collection sites, with couples of neighboring localities being united under the numbers 4 and 6, from 3 provinces of western Argentina. Geographic data and number of cytogenetically studied individuals (N) are as follows: Jujuy province (sites 1–3): Loma Blanca (N=2), 22°26'30.25"S; 66°26'28.57"W, Abra Pampa (N=1), 22°46'4.80"S; 65°42'10.08"W, Susques (N=1), 23°23'8.88"S; 66°32'15.72"W. Catamarca province (site 4): Cortaderas (N=3), 27°35'3.84"S; 68°8'57.12"W and Pastos Largos (N=1), 27°40'8.40"S; 68°9'36.00"W. Mendoza province (sites 5–7): Uspallata (N=1), 32°46'30.01"S; 69°36'14.39"W, Las Heras (N=2), 32°49'12.00"S; 69°65'52.79"W and Quebrada del Toro (N=1), 32°31'12.00"S; 69°0'36.01"W, Malargüe (N=10), 36°4'26.40’’S; 69°32'2.40"W.

Animals were captured using Sherman traps and the voucher specimens are housed in the mammal collection of the Instituto Argentino de Zonas Áridas (CMI – IADIZA), CCT-Mendoza, CONICET. Catalog numbers of studied specimens correspond to the Colección Mastozoológica del IADIZA: CMI. Provincia de Jujuy: Localidad Loma Blanca (07508, 07509); Abra Pampa (006998); Susques (006999). Provincia de Catamarca: Localidad Cortaderas (007132, 007134, 007177); Pastos Largos (007186). Provincia de Mendoza: Localidad Uspallata (007395), Las Heras (007391, 007398); Quebrada del Toro (006797); Malargüe (006794, 006792, 006791, 006790, 007400, 07505, 07506, 07422, 07421, 07507).

Mitotic chromosome preparations were obtained from bone marrow using the traditional cell suspension technique (Ford and Hamerton 1956). Chromosomes were stained with Giemsa (pH=6.8). Ten metaphase spreads were counted for each specimen. The distribution of constitutive heterochromatin (C-bands) was determined according to the Sumner’s (1972) method. The technique of Schweizer (1978, 1980) was used for CMA_3_/DAPI staining. Photomicrographs were obtained using an Olympus BX 50 photomicroscope, with a Sony Exwave Had digital camera.

## Results

### Solid staining

Karyotypes of all individuals of *Phyllotis xanthopygus* analyzed had 2n=38, with 18 autosomal pairs which can be arranged by decreasing size, and a pair of XY sex chromosomes. Most of the autosomal complement was characterized by meta-submetacentric chromosomes. But, due to the presence of chromosome heteromorphisms, the fundamental number of autosomal arms (FNa) varied between 70 and 72. The X chromosome is one of the largest elements and the Y chromosome one of the smallest, both metacentric.

**Figure 2.**
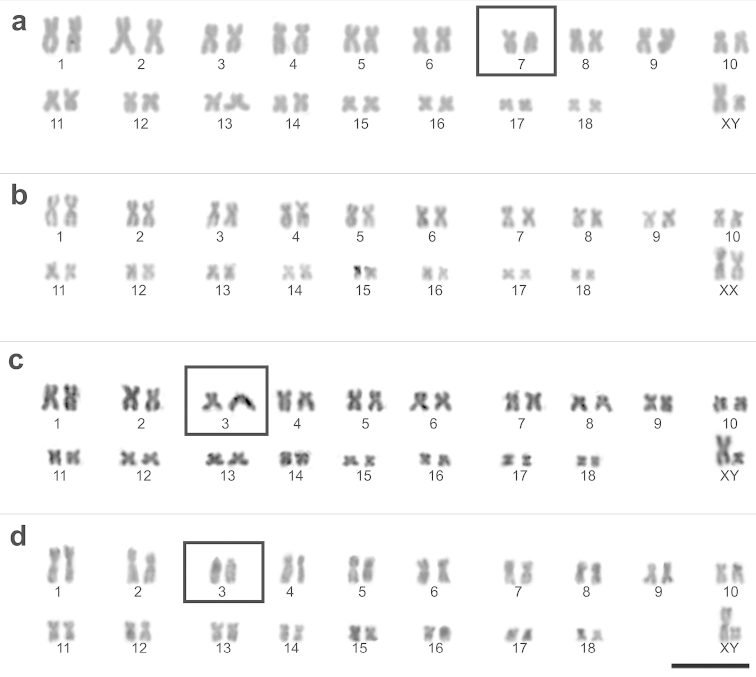
Karyotype variation in *Phyllotis xanthopygus*, 2n=38, from provinces: Mendoza, (**a** site 7), FNa=71; Catamarca (**b** site 4), FNa=72; Jujuy (**c** sites 1 and 3) FNa=71 and (**d** sites 1 and 2) FNa=70. Chromosome heteromorphisms are in boxes. Routine Giemsa staining. XX, XY – sex chromosomes. Bar = 10 µm.

In Malargüe, southern Mendoza province, two different karyotypes were observed. Four individuals (two females and two males) had FNa=72 with all chromosomes biarmed, and another six specimens (two females and four males) had FNa=71 with one heteromorphic pair (number 7 when arranged by size) composed of one acrocentric and one submetacentric chromosome (Fig. 2a). Individuals from northern Mendoza (four males) and Catamarca (three females and one male) provinces showed karyotypes composed entirely of biarmed chromosomes, with FNa=72 (Fig. 2b). In Jujuy province, two individuals (two males) had FNa=71, with one heteromorphic pair (number 3 when arranged by size) composed of one acrocentric and one submetacentric chromosome (Fig. 2c). The remaining two specimens (one female and one male) had FNa=70, with two acrocrocentric chromosomes (pair 3). In one of these acrocentric chromosomes, it was possible to distinguish a small chromosome arm (Fig. 2d).

### C-banding

**Figure 3.**
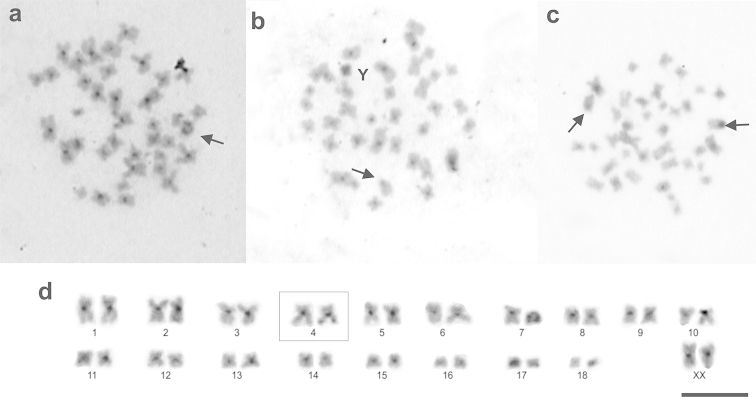
C-banding pattern in metaphase chromosomes of *Phyllotis xanthopygus*. **a** Female from Malargüe, the presence a large block of heterochromatin in acrocentric chromosome (arrow) **b** Small pericentromeric C-band in an acrocentric autosome (arrow), and the entirely heterochromatic Y chromosome, Jujuy, male **c** Small C-band in one acrocentric and a prominent pericentromeric C-heterochromatin in the second acrocentric, Jujuy, female (arrows) **d** C-banded karyotype, with small telomeric C-bands in one autosome pair (in box) and the heteromorphic metacentric/acrocentric pair No.7. Bar = 10 µm.

In the specimens studied, positive C-bands were observed in the pericentromeric regions of all chromosomes (Fig. 3). Additionally, very small telomeric C-bands can be detected in some autosomes (Fig. 3d). The X chromosome was indistinguishable from the autosomal complement with respect to the amount of CH, while the Y chromosome was completely C-positive (Fig. 3b).

Different amounts of CH were observed in the acrocentric chromosome of individuals from the south and north of the country. In Malargüe (south of Mendoza province), the acrocentric chromosome was almost completely heterochromatic (Fig. 3a, d). In the north, in Jujuy, we observed two heterochromatic variants of the acrocentrics. One had very tiny pericentromeric C-bands (Fig. 3b) and the other one had a large C-band around the centromere. Specimens with only one acrocentric had the variant with small CH (Fig. 3b), while those with two acrocentrics carried both variants (Fig. 3c).

### Fluorochromes

**Figure 4.**
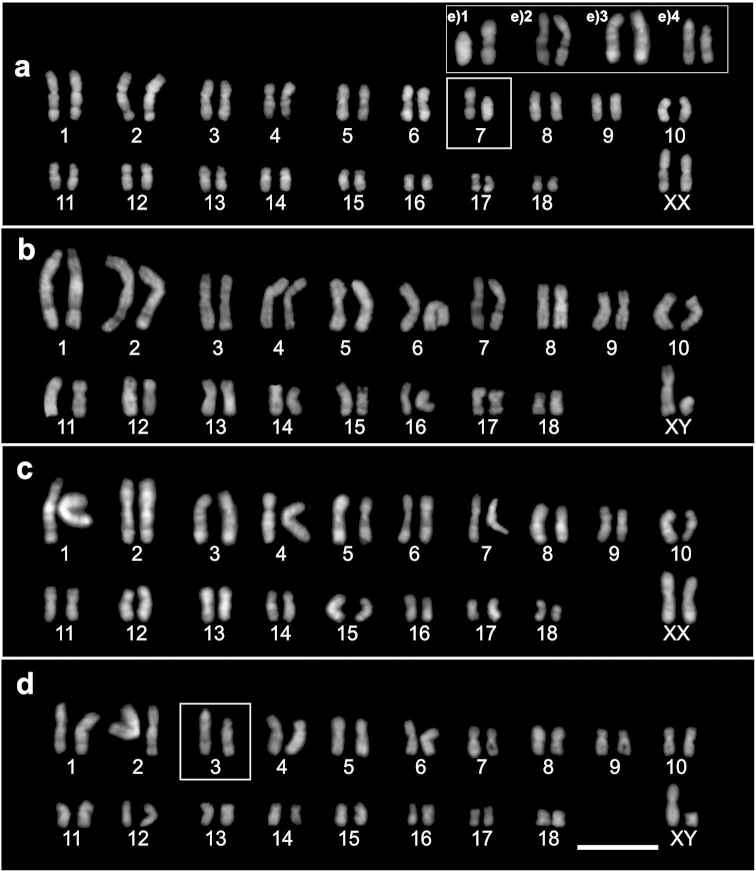
The DAPI staining of chromosomes of *Phyllotis xanthopygus* revealing: **a** Heteromorphic pair 7, female from Malargüe, the south of Mendoza **b** Homozygous metacentrics of male from the north of Mendoza **c** Homozygous metacentrics of female from Catamarca Province **d** Heterozygous pair 3 in male from Jujuy. In boxes are the heteromorphic pairs **e** Details of pairs involved in the chromosome polymorphisms described in this work: **e)1** pair 7 from south Mendoza population, **e)2** pair 7 from north of Mendoza, **e)3** pair 3 from Catamarca, **e)4** pair 3 from Jujuy, the size of chromosomes was modified for a better comparison of DAPI bands. Bar = 10 µm.

**Figure 5.**
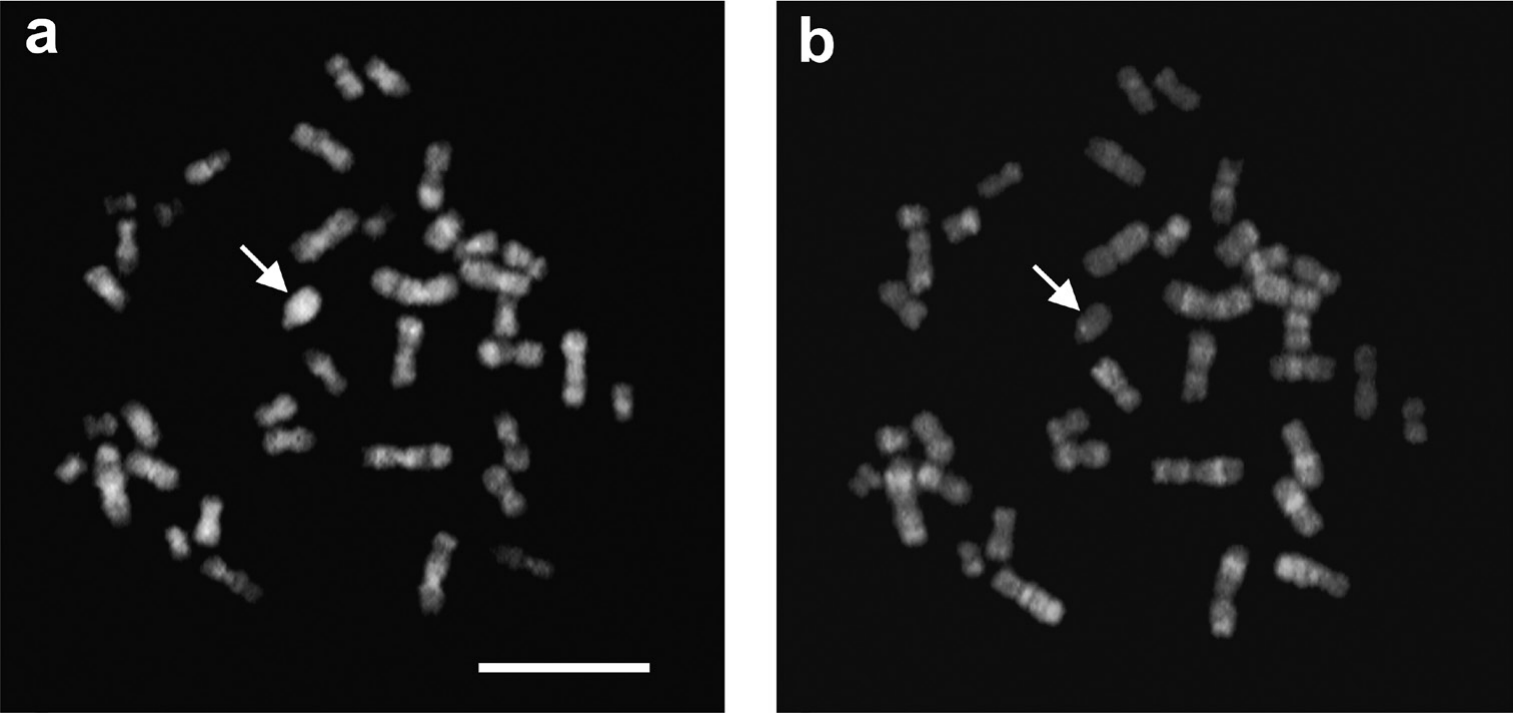
An acrocentric of a heteromorphic pair No. 7 from a metaphase fig treated with DAPI (**a**) and CMA_3_ (**b**). Bar = 10 µm.

The DAPI bands of *Phyllotis xanthopygus* revealed similar localization among the karyotypes of specimens from different geographic regions (Fig. 4a–d). We found homology in most autosomal pairs. However, pairs such as 12, 14 and 15, have not been seen completely homologous particularly in karyotypes from Jujuy. This can be due to differences in chromosome condensation, or to small chromosome rearrangement not detected with the cytogenetic techniques used in this work. The pericentromeric regions of nearly all chromosomes appeared positive with DAPI and negative or neutral with CMA_3_ (Fig. 5).

On the other hand, within the heteromorphic pair 7 from Malargüe, the acrocentric chromosome presented a large DAPI-positive/CMA_3_-neutral block, which covered almost the entire chromosome length, and a small DAPI-negative/CMA_3_-positive block in the pericentromeric region (Fig. 5). The biarmed chromosome of this pair showed DAPI-negative blocks in the interstitial region of the long arm (Fig. 4). We found great homology between the biarmed chromosomes of pair 7 from southern (Malargüe) and northern Mendoza (Fig. 4). Among the chromosomes of specimens from Catamarca, pair 3 appears to be homologous to the biarmed chromosome of the heteromorphic pair 7 from Malargüe (Fig. 4). In Jujuy, the acrocentric homologs of pair 3 contained an interstitial DAPI-negative/CMA_3_-neutral block in their long arm. Besides, while one acrocentric chromosome showed a DAPI-positive band in the centromeric region, this band was absent in the other one. Therefore, the homology between both acrocentric chromosomes is only partial (Fig. 4).

With respect to sex chromosomes, the X chromosome revealed the pericentromeric region as DAPI positive/CMA_3_-neutral. Additionally, a large DAPI-positive/CMA_3_-neutral band was evidenced in the telomeric region of its long arm. The Y chromosome was found to be almost completely fluorescent with DAPI staining in all specimens analyzed (Fig. 4).

## Discussion

Although *Phyllotis xanthopygus* is one of the widely distributed rodents across the countries situated along the Andean landscapes, the range of the karyotype variation in this species is rather partially known. In our work, samples studied in Argentina add new cytogenetic information on this species. The chromosome complement of 2n=38 is found all over its distribution area, including the territories of the anew involved in chromosome examination provinces of Jujuy, Catamarca and Mendoza by western boundary of the country. It is confirmed that the species karyotype is composed almost exclusively of biarmed-metacentric and submetacentric-chromosomes that corresponds to previous reports for this taxon and for some other related species (Pearson and Patton 1976, Walker et al. 1991, 1999, Kramer et al. 1999).

Studies of chromosome homologies in Argentine specimens of *Phyllotis xanthopygus* have not yet been performed and might be of interest due to the commonly expected cytogenetic input in the establishment of taxonomic identity and chromosome relations of geographic populations. The chromosome banding pattern obtained in this work using DAPI staining is largely comparable to the G-band pattern published for the three subspecies *Phyllotis xanthopygus vaccarum*, *Phyllotis xanthopygus rupestris* and *Phyllotis xanthopygus xanthopygus* (Walker et al. 1991). G-banding patterns were similar among the chromosomes of these three subspecies of *Phyllotis xanthopygus* analyzed by Walker et al. (1991), with the exception of the sex chromosome from *Phyllotis xanthopygus xanthopygus*. The G-banding pattern for the X chromosome in the latter subspecies had correspondence to that observed for the same chromosome in our work with DAPI banding. These results indicate that our specimens are chromosomally more similar to *Phyllotis xanthopygus xanthopygus* than to any other subspecies, at least in euchromatic regions.

The specific separation of *Phyllotis darwini* and *Phyllotis xanthopygus* is well supported (Spotorno and Walker 1983, Walker et al. 1984). Accordingly, when we compared the pattern obtained for our DAPI-stained sample with the G-banding pattern of *Phyllotis darwini*, we observed chromosomal differentiation in several chromosome pairs, similar to that previously described by Walker et al. (1984).

Constitutive heterochromatin (CH) is a feature that is often variable among the karyotypes of mammals showing different patterns in members of the genus *Phyllotis* as in different taxa of mammals (Walker et al. 1991, Graphodatsky et al. 2011). The pericentromeric location of CH is a predominant characteristic in *Phyllotis* and in other rodents (Patton and Sherwood 1983). However, variations in the amount of CH have been demonstrated for the subspecies of *Phyllotis xanthopygus*. The pattern of CH obtained in this study is not completely consistent with those described for the subspecies of *Phyllotis xanthopygus* analyzed in other studies. Most autosomes of *Phyllotis xanthopygus xanthopygus* exhibited very tiny pericentromeric C-bands or a few small ones, with exception of pair 15, which showed a larger one. On the other hand, *Phyllotis xanthopygus rupestris* and *Phyllotis xanthopygus vaccarum* presented large pericentromeric C-bands in all autosomes (Walker et al. 1984, 1991, 1999).

The Y chromosome was completely C-positive in all populations analyzed. The same pattern was identified in the subspecies *Phyllotis xanthopygus rupestris* and in *Phyllotis darwini*. However, as mentioned above, the autosomal C-band pattern in *Phyllotis xanthopygus rupestris* subspecies does not correspond to those obtained in this study. In subspecies *Phyllotis xanthopygus vaccarum* and *Phyllotis xanthopygus xanthopygus* the whole Y chromosome was faintly heterochromatic (Walker et al. 1991).

Despite the uniformity in most chromosomes of the complement, we found intra and inter-population variations, which resulted in modifications of the FNa from 70 to 72. In Malargüe we observed high frequency of individuals with a heteromorphic pair (FNa=71). Also in Jujuy province we observed chromosome heteromorphisms. In this last region, Pearson and Patton (1976) described specimens with a single acrocentric chromosome. In our sample from Jujuy, we also observed specimens with two different acrocentric chromosomes (FNa=70). In the north of Mendoza and in Catamarca we found no acrocentric chromosomes, but this could be because of the small size of the samples from these geographically intermediate areas (Fig. 1).

Additionally, we found differences in the quantity and distribution of CH when comparing the acrocentric chromosomes within and among localities. In Malargüe, this chromosome is almost completely heterochromatic. The absence of homozygous individuals with acrocentric chromosomes in this locality could be due small sample size. Alternatively this chromosomal condition could be negatively heterotic, since large additions of heterochromatin are probability related to loss of gene function and genetic degeneration (John 1988, King 1993, Waters et al. 2007).

A geographic variation of heterochromatin is shown in this work. In Jujuy province, in the north of the country, acrocentric chromosomes showed much less amount of CH than in the south in Malargüe. At the same time, two different acrocentric chromosomes varying in morphology and in the amount of heterochromatin were detected in Jujuy specimens. It can be suggested that this variation in amount of CH is due to a gradual process of heterochromatin addition or deletion in these chromosomes. But additional evidence in the sequences involved is necessary to confirm this hypothesis.

Application of fluorochromes also allowed us to study the possible structural rearrangements that generated the south to north variation in FNa. The acrocentric chromosome from Malargüe showed no homology with any other chromosome of complement, but the biarmed chromosome of the pair showed high homology with pair 7 from northern Mendoza and with pair 3 from Catamarca and Jujuy. In addition, the two different acrocentric chromosomes of pair 3 from Jujuy showed partial homology via fluorochromes (Fig. 4). These results can only be explained by a complex sequence of rearrangements, possibly involving amplifications and/or deletions of heterochromatic segments. Karyotype variation in the amount of heterochromatin within and among populations are common in some rodents species as *Mus musculus*, *Perognathus baileyi*, etc. (Patton and Sherwood 1983, Graphodatsky et al. 2011).

The role of chromosomal changes in the differentiation of populations and speciation has been the subject of continued interest and controversy (Patton and Sherwood 1983, Faria and Navarro 2010, Romanenko and Volobouev 2012, Romanenko et al. 2012). Cytogenetic data showed moderate chromosome variability and differentiation within and between populations. These results are consistent with previous works on other Sigmodontinae species that also show great chromosomal variability at intra- and inter-population level (Patton and Sherwood 1983, Lanzone et al. 2011). However, considerable additional data will be required to clarify the taxonomic status of *Phyllotis xanthopygus* and its subspecies, as well as to understand the evolutionary process that generates this diversity.
